# Programmable nanoengineering templates for fabrication of three-dimensional nanophotonic structures

**DOI:** 10.1186/1556-276X-8-268

**Published:** 2013-06-07

**Authors:** Qingfeng Lin, Siu-Fung Leung, Kwong-Hoi Tsui, Bo Hua, Zhiyong Fan

**Affiliations:** 1 Department of Electronic and Computer Engineering, Hong Kong University of Science and Technology, Clear Water Bay, Kowloon, Hong Kong SAR, China

**Keywords:** Large-pitch anodic alumina membranes, Programmable nanoengineering templates, Nanocones, Three-dimensional nanophotonic structures

## Abstract

Porous anodic alumina membranes (AAMs) have attracted great amount of attention due to their potential application as templates for nanoengineering. Template-guided fabrication and assembly of nanomaterials based on AAMs are cost-effective and scalable methods to program and engineer the shape and morphology of nanostructures and nanomaterials. In this work, perfectly ordered AAMs with the record large pitch up to 3 μm have been fabricated by properly controlling the anodization conditions and utilization of nanoimprint technique. Due to the capability of programmable structural design and fabrication, a variety of nanostructures, including nanopillar arrays, nanotower arrays, and nanocone arrays, have been successfully fabricated using nanoengineered AAM templates. Particularly, amorphous Si nanocones have been fabricated as three-dimensional nanophotonic structures with the characterization of their intriguing optical anti-reflection property. These results directly indicate the potential application of the reported approach for photonics and optoelectronics.

## Background

The capability to program and engineer the shape and morphology of nanostructures and nanomaterials enables tailoring their electronic [1-3], optical [4-6], sensing [7,8], thermal [9,10], and mechanical [11-14] properties for a variety of applications including electronics, photovoltaics, sensors, thermoelectrics, nanomechanical devices, etc. Specifically, a variety of three-dimensional (3-D) nanophotonic structures, such as nanowires [15,16], nanopillars [17,18], nanowells [19], and so forth, have been extensively studied for efficient light harvesting scheme to enhance the performance of solar cells. Properly engineered 3-D nanostructures have demonstrated highly promising capability of harvesting sunlight over a broad range of wavelengths and incident angles due to their broadband anti-reflection and efficient light trapping properties. On the other hand, cost-effective approaches toward the precise control of the shape and morphology of nanostructures are crucial for any aforementioned practical applications. In general, nanofabrication methods used to produce nanostructures are commonly defined as ‘top-down’ and ‘bottom-up’ methods [20]. The top-down approaches, which use various kinds of lithographic techniques to pattern nanoscale structures typically in two dimensions, allow to fabricate different and complex structures with high precision. However, their major disadvantage rests in high cost and limited scalability. Conversely, the bottom-up approaches, which utilize energetic favorable self-assembly and/or self-organizing mechanisms to form nanostructures, are cost-effective but usually lack of controllability over as-obtained macro- and nanostructures. In this regard, a cost-effective and scalable method combining the advantages of both top-down and bottom-up approaches will be highly appealing. Template-guided fabrication and assembly of nanomaterials, particularly based on porous anodic alumina membranes (AAMs), have attracted great amount of attention due to the fact that AAMs are formed by self-organizing mechanism and they enable direct assembly of nanomaterials with controlled density [21-25]. Meanwhile, recent achievements on controlling template regularity and internal structure clearly demonstrate their potency for the precise integration of nanomaterials with high degree of freedom [17,26-28]. In this work, we present the fabrication of AAMs with perfect regularity and unprecedented large pitch up to 3 μm by applying high-voltage anodization in conjunction with nanoimprint process. More importantly, due to the capability of programmable structural design and fabrication, a variety of nanostructures, including nanopillar arrays, nanotower arrays, and nanocone arrays, have been successfully fabricated using nanoengineered AAM templates. Particularly, the nanocone arrays have been demonstrated as excellent 3-D nanophotonic structures for efficient light harvesting due to the gradually changed effective refractive index.

## Methods

## Materials

Aluminum foil (0.25 mm thick, 99.99% purity) was obtained from Alfa Aesar (Ward Hill, MA, USA), polyimide solution (PI 2525) was purchased from HD MicroSystems (Parlin, NJ, USA), polycarbonate film (0.2 mm thick) was obtained from Suzhou Zhuonier Optical Materials Co., Ltd. (Suzhou, China), epoxy glue (Norland Optical Adhesive 81) was purchased from Norland Products Inc. (Cranbury, NJ, USA), silicone elastomer and the curing agent were purchased from Dow Corning (Midland, MI, USA). All other chemicals are products of Sigma-Aldrich (St. Louis, MO, USA).

### AAM fabrication

**Figure 1:**
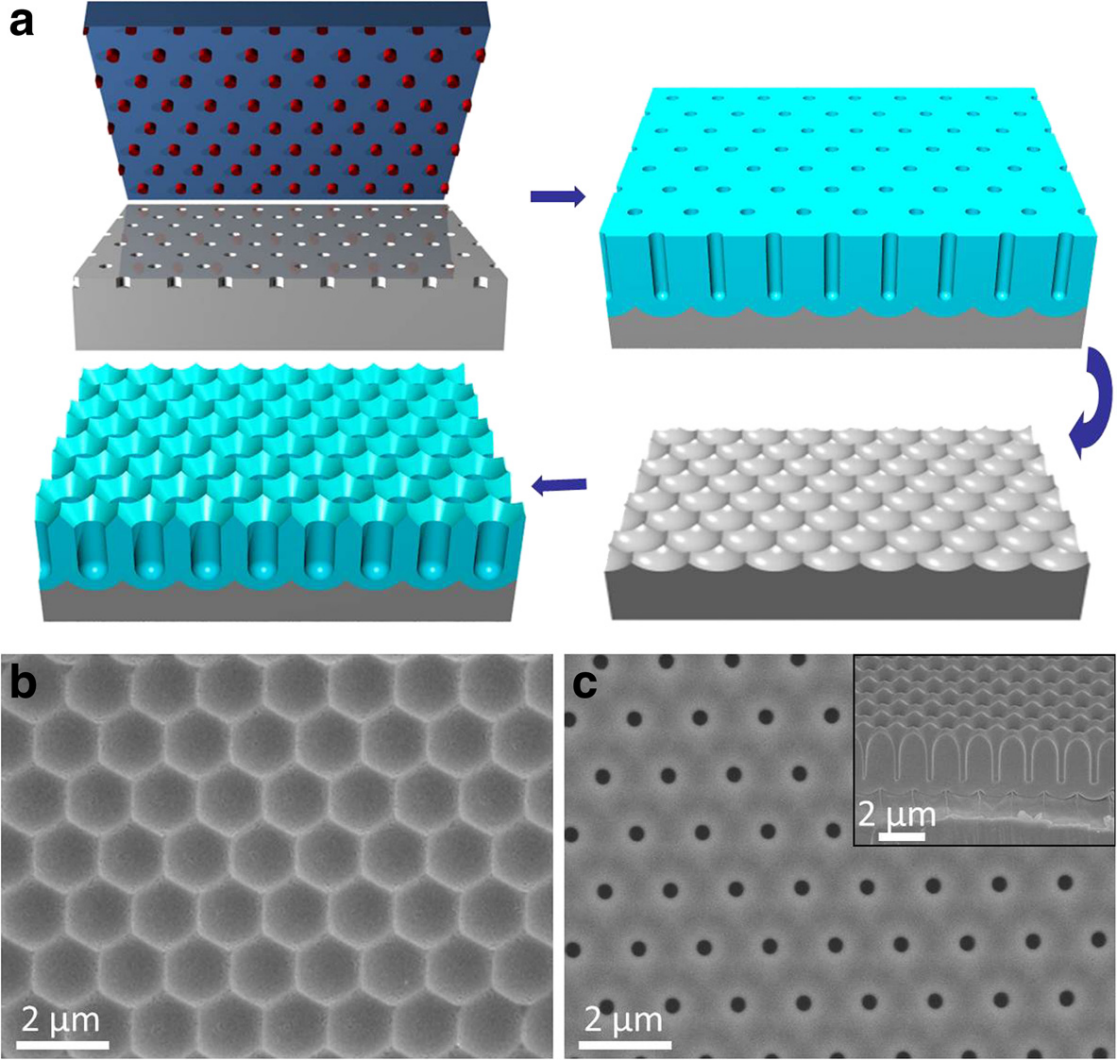
**Schematic fabrication process and top-view scanning electron microscopy (SEM) images of AAM.** (**a**) Schematic fabrication process of hexagonally ordered porous AAM. (**b**) Top-view SEM image of 1.5-μm-pitch Al concave structure after the removal of the first anodization layer. (**c**) Top-view SEM image of 1.5-μm-pitch AAM after the second anodization, with the cross-sectional view showing cone-shape opening in the inset.

Aluminum (Al) foil was cut into 1-cm by 2-cm pieces and cleaned in acetone and isopropyl alcohol. The sheets were electrochemically polished in a 1:3 (*v*/*v*) mixture of perchloric acid and ethanol for 2 min at 10°C. As shown in Figure 1a, the polished Al sheets were imprinted using silicon mold (hexagonally ordered pillar array with height of 200 nm, diameter of 200 to 500 nm, and pitches ranging from 1 to 3 μm) with a pressure of approximately 2 × 10^4^ N cm^−2^ to initiate the perfectly ordered AAM growth. The substrates were anodized with conditions listed in Table 1. The first anodization layer was then etched away in a mixture of phosphoric acid (6 wt.%) and chromic acid (1.8 wt.%) at 63°C for 40 min. After etching, the second anodization was carried out under the same conditions to obtain approximately 2-μm-thick AAM. Afterward, desired pore diameters of AAMs were obtained by wet etching with 5% phosphoric acid at 53°C. In order to achieve tri-diameter AAM, a substrate was secondly anodized for time *t*_A1_ using the same anodization conditions and etched in 5% phosphoric acid at 53°C for *t*_E1_ to broaden the pores and form the large-diameter segment of the membrane. Then, the third anodization step at the same condition was performed for another time *t*_A2_ followed by phosphoric acid etch for time *t*_E2_ to form the medium-diameter segment of the pore. In the end, the fourth anodization step was carried out at the same condition for time *t*_A3_ ending with time *t*_E3_ wet etching to form the small-diameter segment of the membrane. Note that in this scenario, the large-diameter segment was etched for *t*_E1_ + *t*_E2_ + *t*_E3_ to broaden the pore size close to the pitch of the membrane, and the diameters can be determined using the plot shown in Additional file 1: Figure S1a,b.

**Table 1:** Anodization conditions of perfectly ordered large pitch porous AAMs

**Pitch (μm)**	**Voltage (V)**	**Temperature (°C)**	**Solution**
1	400	10	230 mL, 1:1, 4 wt.% citric acid/ethylene glycol (EG) + 15 mL 0.1% H_3_PO_4_
1.5	600	2	240 mL, 1:1, 1 wt.% citric acid/EG + 1.5 mL 0.1% H_3_PO_4_
2	750	3.2	240 mL, 1:1, 0.1 wt.% citric acid/EG
2.5	1,000	2	240 mL, 1:1, 0.05 wt.% citric acid/EG
3	1,200	2	240 mL, 1:1, 0.05 wt.% citric acid/EG

### PI nanopillar array assembly

Six hundred microliters of PI solution was dispensed on an AAM substrate. After tilting and rotating the substrate to spread the solution to achieve full substrate coverage, the substrate was spin-coated on a spin-coater (Model WS-400BZ-6NPP/LITE, Laurell Technologies Corporation, North Wales, PA, USA) at 500 rpm for 30 s first, then quickly accelerated (2,000 rpm/s) to 1,000 rpm for 30 s. After spin-coating, the substrate was transferred to a hot plate to cure PI solution, started from room temperature to 300°C with a ramping rate of 20°C/min, and maintained at 300°C for 10 min. The cured substrate was then bonded to a PC film with epoxy glue, then cured by a 4-W UV lamp (Model UVL-21 Compact UV lamp, UVP, LLC, Upland, CA, USA) for 10 h. In the end, PI nanopillar arrays were transferred to the PC film by directly peeling off the PC film from the AAM substrate.

### Bonding of the a-Si nanocones device on glass and PDMS

The AAM substrate with amorphous silicon (a-Si) nanocone array deposition was attached to a glass slide with epoxy glue, then cured by a 4-W UV lamp for 10 h. The Al substrate was etched from the back side in a saturated HgCl_2_ solution, followed by removal of AAM in HF solution (0.5 wt.% in deionized water) with high selectivity over a-Si nanocone array. For the mechanically flexible device, instead of glass, polydimethylsiloxane (PDMS) was used for the encapsulation. To encapsulate the device with PDMS, silicone elastomer was mixed with the curing agent (10:1 weight ratio) at room temperature, then poured onto the device in a plastic dish to form an approximately 2-mm layer, and cured at 60°C for 6 h. The Al substrate and AAM were then removed sequentially by the aforementioned etching process. Finally, approximately 2-mm-thick PDMS was cured on the back side of the substrate to finish the encapsulation process.

### Microscopy and spectroscopy

Scanning electron microscopy (SEM) images of uncoated or Au-coated samples were obtained using a JEOL JSM-6700 F SEM (JEOL Ltd., Akishima-shi, Japan) working at 5 kV. Ultraviolet–visible (UV–vis) spectra of all samples were recorded on a Perkin Elmer Lambda 20 UV/Vis Spectrometer (Perkin Elmer, Waltham, MA, USA). Finite-difference time-domain (FDTD) simulation was employed to confirm the reflection property of the nanocone arrays as fabricated in the experiments.

## Results and discussion

Electrochemical anodization of aluminum (Al) in acidic solution to form porous alumina has been well documented [29-31]. The self-organizing mechanism typically yields nanopore arrays with a few micrometers short range hexagonal ordering [32-34]. As the process is facile and low cost, it has been widely used for assembly of nanowires and nanotubes previously [17,21,25-27]. Meanwhile, Masuda et al. has reported fabrication of long-range perfect-ordered AAM with pitch less than 500 nm by texturing Al surface [35]. On the other hand, in order to fabricate nanostructures with a wide range of geometries, much larger pitch is required for a number of applications. For example, it has been shown that when photon wavelength is comparable to pitch, it can be efficiently absorbed by the three-dimensional nanowell structure [19]. Therefore, a wide range of pitch enables efficient light-structure interaction for a broad range of wavelength. Nevertheless, perfectly ordered AAM with pitch larger than 500 nm has rarely been reported. The realization of larger pitch was rather challenging due to the ‘breakdown’ or ‘burning’ of the oxide film caused by the catastrophic flow of electric current under higher anodization voltages [36,37]. Recently, we have reported perfectly ordered AAM with pitch up to 2 μm for efficient photon harvesting [19,28]. In this work, we have extended the largest pitch up to 3 μm. The detailed fabrication procedure of hexagonally ordered porous AAM is schematically shown in Figure 1a. Briefly, an Al sheet was polished electrochemically before being imprinted using a Si mold with a hexagonally arranged array of nanopillars, followed by the first anodization with stable high voltage to get ordered anodic alumina channels. The first anodization layer was then etched away (first etch) followed by the second anodization under the same conditions; in this case, the imprinted texture on the top can be removed, leaving the naturally developed porous structure with cone-shape opening. The diameter of the nanopores on the second anodization layer can be controllably widened to desirable size, as shown in Additional file 1: Figure S1a,b. Note that since pitches of structures are larger than 1 μm, the Si imprint molds are fabricated with wafer stepper instead of electron beam lithography [35], thus the molds can be made into large size with high throughput. Figure 1b demonstrates the top-view SEM image of Al concave structure after the removal of the first anodization layer, clearly showing the hexagonal periodicity caused by nanoimprint has been transferred to Al below the first anodization layer of AAM. Figure 1c illustrates the top-view SEM image of perfectly ordered AAM after the second anodization with cone-shape opening, which is easier to be observed from the cross-sectional view, as shown in the inset of Figure 1c.

**Figure 2:**
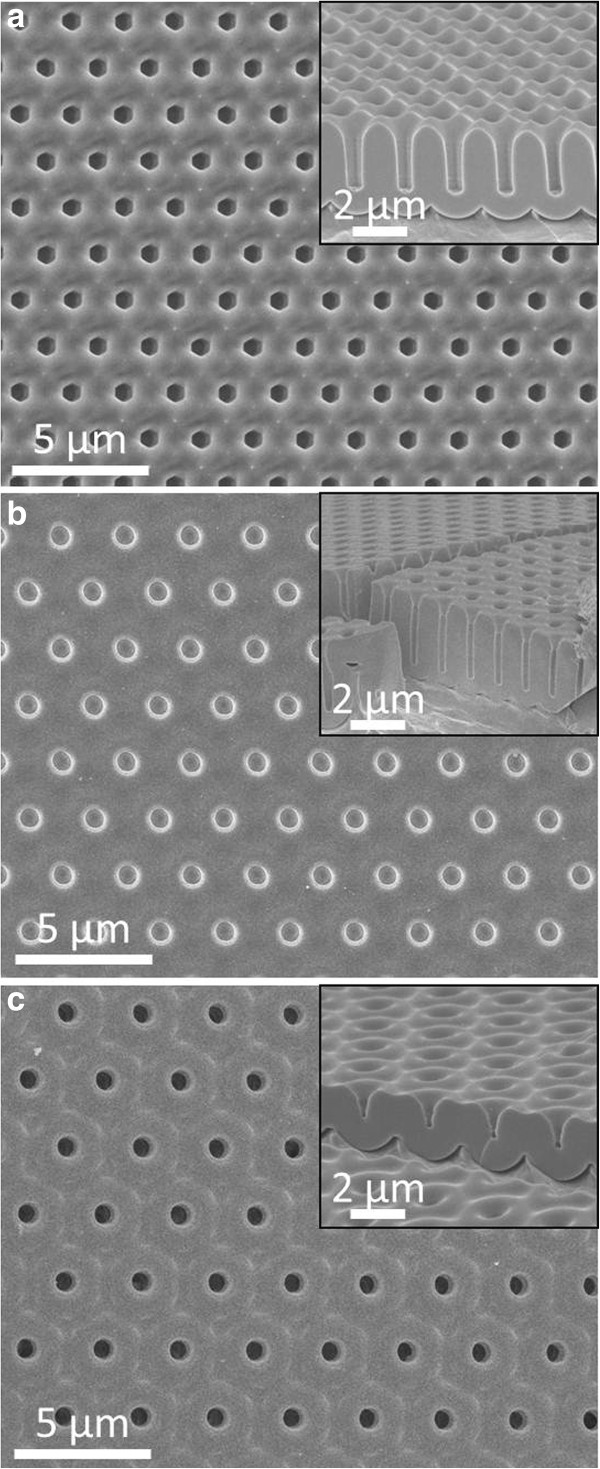
**Top-view SEM images of AAM.** (**a**) Two-micrometer pitch AAM after the second anodization, (**b**) 2.5-μm-pitch AAM after the first anodization, and (**c**) 3-μm-pitch AAM after the first anodization, with their corresponding cross-sectional-view SEM images in the inset.

Beyond AAM with 1.5-μm pitch shown in Figure 1b,c, AAM with much larger pitches including 2-, 2.5-, and 3-μm pitches have also been successfully achieved, as shown in Figure 2. Previous studies indicated that the pitch of AAM fabricated under mild anodization conditions using sulfuric acid, oxalic acid, and phosphoric acid linearly depends on the applied anodization potential with a proportionality about 2.5 nm V^−1^[29-31,36]. Nevertheless, further increase of anodization potentials is limited by the ‘breakdown’ or ‘burning’ of the oxide film caused by the catastrophic flow of electric current under applied high voltages in a given electrolyte solution. It is known that the key factor for achieving perfectly ordered AAM with desired pitch is controlling the balance between the growth and the dissolution of the oxide film by adjusting the acidity, concentration, and temperature of anodization electrolytes [38], as well as modulating the applied voltages around the matching value approximately 0.4 V/nm [36]. Since the pitch of AAM is proportional to the applied anodization potential, high anodization voltage need to be applied to get large-pitch AAM; as a result, the anodization electrolyte should be weak acid to avoid chip burning from occurring. For example, 750-V direct current voltage was applied for anodization of 2-μm-pitch AAM, which is about four times that for 500-nm-pitch AAM (195 V). To maintain the stability of the solution and anodization current, 0.1 wt.% citric acid was used and diluted with ethylene glycol (EG) in 1:1 ratio. Noticeably, it was found that mixing EG with citric acid can further improve the stability of the electrolyte, thus avoid the burning from occurring for anodization with such high voltage [39]. Figure 2a illustrates the top-view SEM image of perfectly ordered 2-μm-pitch AAM after the second anodization, with corresponding cross-sectional-view SEM image shown in the inset. The thickness of AAM can be controlled by modifying the anodization time, and the pore size can be tuned by controlling the etching time.

According to the rationale discussed above, 2.5- and 3-μm-pitch AAMs were also fabricated after hundreds of trials with various anodization conditions. The best anodization conditions of these perfectly ordered large-pitch porous AAMs were summarized in Table 1. Note that as the acids are highly diluted, anodization process occurs in a quite slow rate; therefore, AAM growth rate is very slow as well, as shown in Additional file 1: Figure S1d. Figure 2b,c show SEM images of ordered 2.5- and 3-μm-pitch AAM after the first anodization, instead of after the second anodization. The matching anodization potential for 3-μm-pitch AAM is 1,200 V, which generates massive heat so that the present cooling system was not powerful enough to maintain the temperature stability leading to the burning of oxide films during the anodization process gradually. Therefore, the maximum depth of the channels in 3-μm-pitch AAM after the first anodization we achieved was about 1 μm (inset in Figure 2c). This depth is not sufficient to form deep Al concave texture to guide the self-assembly of porous alumina during the second anodization. Whereas the maximum pitch of ordered porous AAM achieved in this work is up to 3 μm, it is believed that the pitch can be further increased in the future by modifying the anodization conditions more carefully assisted with a more effective cooling system.

**Figure 3:**
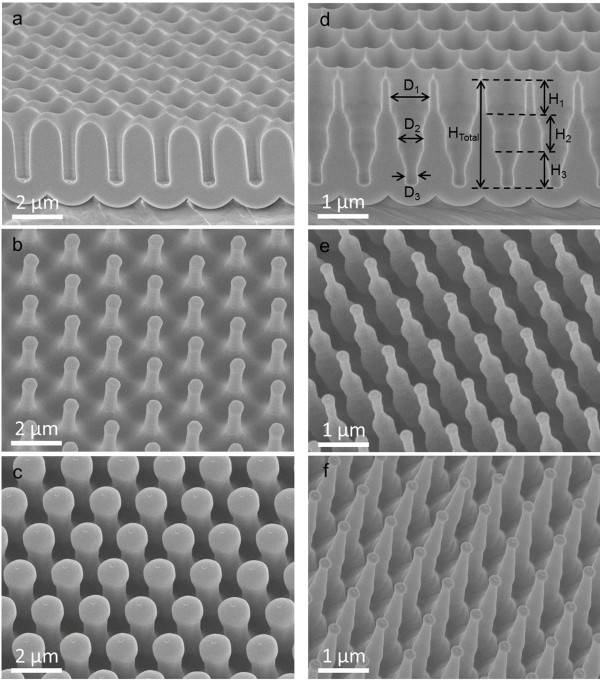
**Cross-sectional-view SEM images of AAM and tilted-view SEM images of PC nanopillar, nanotower, and nanocone arrays.** (**a**) Cross-sectional-view SEM image of 2-μm-pitch AAM with 700-nm pore diameter. The 60°-tilted-angle-view SEM images of (**b**) PC nanopillar arrays templated from 2-μm-pitch AAM with 700-nm pore diameter, and (**c**) PC nanopillar arrays templated from 2-μm-pitch AAM with 1.5-μm pore diameter. (**d**) Cross-sectional-view SEM image of 1-μm-pitch tri-diameter AAM. Tilted-view SEM images of (**e**) PC nanotowers and (**f**) PC nanocones.

As previously mentioned, the fabrication of ordered porous AAMs with hexagonally packed pore arrays has attracted much interest due to their applications as templates for nanoengineering. In fact, we have successfully fabricated nanopillar and nanotower arrays with the large-pitch AAMs, using flexible polymer materials, i.e., polycarbonate (PC) and PI. In order to template PC nanopillars, a PC film was pressed on an AAM on a hot plate with a temperature of 250°C for 15 min to melt PC and fill the AAM channels (Additional file 1: Figure S2a). After cooling down, PC nanopillar arrays were obtained by directly peeling off the PC film from the AAM. Figure 3a shows the SEM image of a 2-μm-pitch AAM with 700-nm diameter for templating PC nanopillars, and Figure 3b illustrates the 60°-tilted-angle-view SEM image of the resulting PC nanopillar arrays with 700-nm pillar diameter. In addition, as the AAM pore diameter can be widened, Figure 3c shows the SEM image of a PC nanopillar array being templated from a 2-μm-pitch AAM with pore diameter of 1.5 μm. Note that the nanopillars shown here have beads on top of them. These beads were formed during peeling process, as shown in Additional file 1: Figure S3.

As the internal structure and shape of AAM nanochannels can be programmed and engineered [17,36], it opens up more degree of freedom on AAM geometry control. In this work, AAMs with three segments with different channel diameters are fabricated by controlling etching and anodization time. Additional file 1: Figure S4 illustrates the schematic process. In brief, a substrate has undergone the second anodization for time *t*_A1_ and etched for *t*_E1_ to broaden the pores and form the large-diameter segment of the membrane. Then, the third anodization step was performed for another time *t*_A2_ followed by chemical etch for time *t*_E2_ to form the medium-diameter segment. In the end, the fourth anodization step was carried out for time *t*_A3_ ending with time *t*_E3_ wet etching to form the small-diameter segment. Note that in this scenario the first segment (Figure 3d) was etched for time *t*_E1_ *+ t*_E2_ *+ t*_E3_, and the third segment was etched only for *t*_E3_ to broaden the pore size. In a generalized case, if there are *n* segments in total, the total etching time for the *m*th segment will be $T_{\mathrm{Em}}=\overset{n}{\underset{i=m}{\sum }}t_{\mathrm{Ei}}$. Therefore, the diameter of the *m*th segment can be determined by the etching calibration curve and the fitted function (Additional file 1: Figure S1a,b) $D_{m}=F\left(T_{\mathrm{Em}}\right)=F\left(\overset{n}{\underset{i=m}{\sum }}t_{\mathrm{Ei}}\right)$. In addition, the total depth of the AAM substrate is $H_{\mathrm{Total}}=\overset{n}{\underset{i=1}{\sum }}H_{i}$ with the *m*th segment's depth of *H*_*m*_ = *G*(*t*_A*m*_) which can be determined by the plots shown in Additional file 1: Figure S1c,d. Figure 3d demonstrates the cross section of a 1-μm-pitch tri-diameter AAM fabricated by a four-step anodization process. Such a structure has been used to template PC nanotowers, as shown in Figure 3e,f, by the aforementioned thermal press process (Additional file 1: Figure S2b). Note that as the length of each diameter segment is controllable, a smooth internal slope on the side wall can be achieved by properly shortening each segment. Therefore, a nanocone structure can be obtained, as shown in Figure 3f.

**Figure 4:**
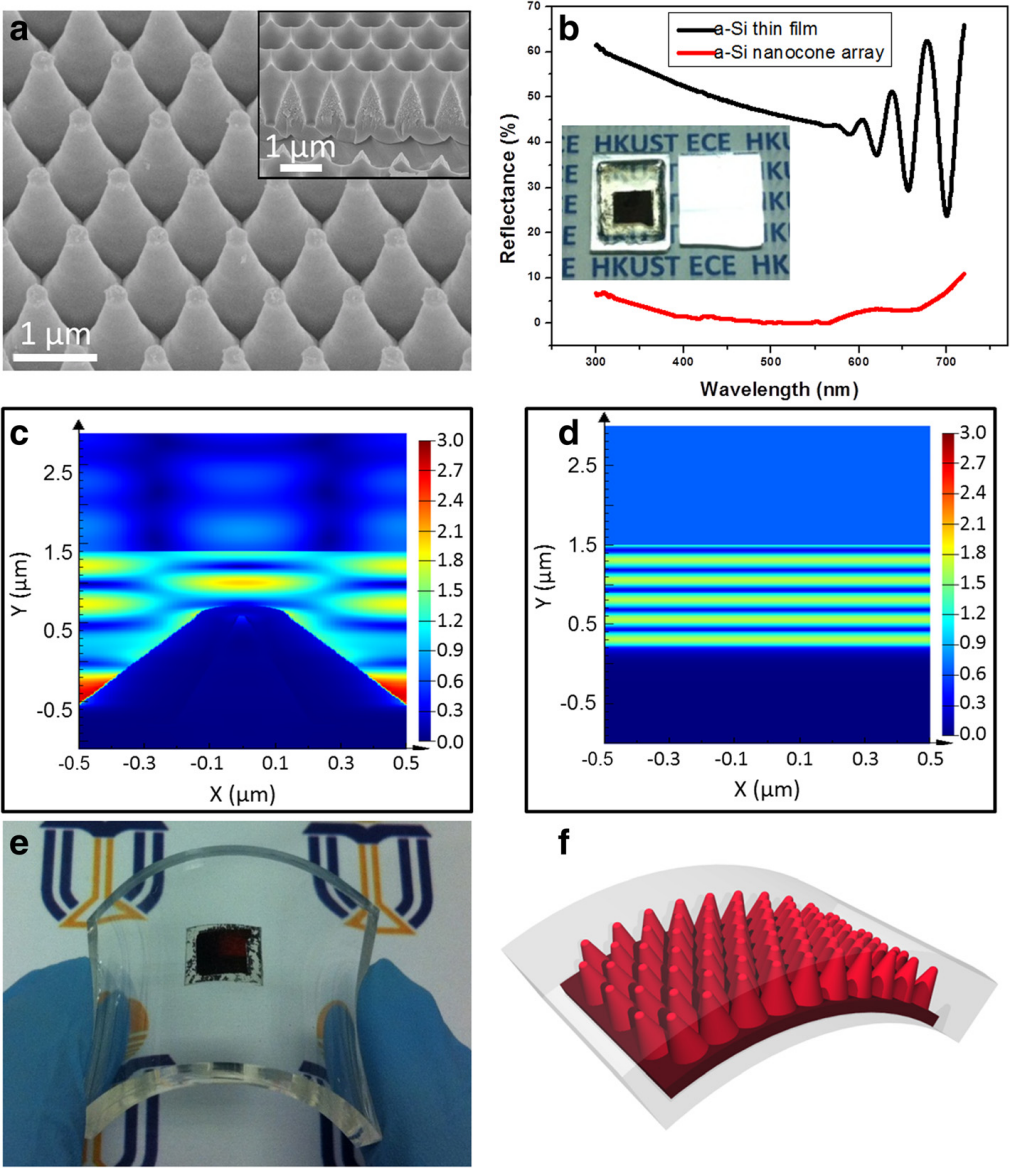
**SEM image, optical reflectance, and photo/schematic of a-Si and cross-sectional |*E*| distribution of the electromagnetic (EM) wave.** (**a**) The 60°-tilted-angle-view SEM image of amorphous Si (a-Si) nanocone arrays fabricated with plasma-enhanced chemical vapor deposition (PECVD), with the AAM template shown in the inset. (**b**) Optical reflectance of a-Si nanocone array and a-Si thin film, with the photographs of a-Si nanocone array (left photo) and a-Si thin film (right photo) on a transparent glass substrate shown in the inset. (**c**, **d**) Simulated cross-sectional |*E*| distribution of the EM wave on nanocone arrays and planar. (**e**, **f**) Photo and schematic of flexible a-Si nanocone array embedded in PDMS.

It is worth noting that the above nanostructure templating process can be extended to other materials. In practice, we have also fabricated PI nanopillar arrays (Additional file 1: Figure S3) with spin-coating method. Besides using thermal press method to template nanostructures, material deposition method was also used to fabricate well designed nanostructures with AAM. Particularly, a-Si nanocone arrays have been fabricated with plasma-enhanced chemical vapor deposition (PECVD), as shown in Figure 4a with the inset showing the AAM template. The nanocones are formed by a-Si thin-film deposition. Additional file 1: Figure S5 shows the cross section of the a-Si nanocones embedded in the AAM. In order to characterize the nanocones, they are transferred to a supporting substrate followed by etching away the AAM template in HF solution.

It is noteworthy that the nanocone structure is a highly promising structure for efficient light harvesting, due to the gradually changed effective refractive index, thus it has been used for improving performance of solar cells [40-42]. In this work, optical reflectance of a-Si nanocones was characterized and shown in Figure 4b. As shown in the inset of Figure 4b, 1-μm-pitch a-Si cone array on a transparent glass substrate shows black color with very low reflectance, as a comparison, a-Si thin film on the glass substrate deposited at the same time with PECVD appears to be mirror-like specular reflective. To further characterize optical properties of the a-Si nanocone array, its optical reflectance was measured with UV–vis spectroscopy equipped with an integrating sphere, together with the a-Si thin film deposited on glass for comparison. As shown in Figure 4b, the a-Si thin film on planar glass demonstrates 25 to 65% high reflectance with wavelength below 720 nm corresponding to a-Si band-gap. In contrast, the a-Si cone array has below 10% reflectance within the same wavelength range, with the minimum reflectance less than 1% at 500-nm wavelength, corresponding to peak of solar irradiance spectrum. In order to corroborate the experimental results, as well as to gain insight into the light propagation in the structures, FDTD simulations were performed on these two structures at 500-nm wavelength, with the cross-sectional electric field intensity (|*E*|) distribution of the electromagnetic (EM) wave plotted in Figure 4c. In the simulations, EM plane waves propagate downward from *Y* = 1.5 μm. Note that the color index at the specific location in the simulations reflects the magnitude of |*E*| at that point, normalized with that of the source EM wave if propagating in free space. It can be observed that a-Si nanocone array demonstrates quite low reflectance, indicated by the small magnitude of |*E*| above *Y* = 1.5 μm (Figure 4c). On the contrary, a-Si planar structure shows much higher reflectance (Figure 4d). Low reflectance of a-Si nanocone array indicates an efficient light absorption in the structure, which is attributed to the gradual change of its effective refractive index. In addition, as the supporting substrate can be arbitrary, flexible PDMS substrates were used and demonstrated in Figure 4e, with the schematic device structure shown in Figure 4f. This result clearly shows the promising potency of the fabricated large-pitch AAM as three-dimensional flexible template for efficient photovoltaics.

## Conclusions

In summary, we have demonstrated fabrication of perfectly ordered AAM with the record large pitch up to 3 μm by properly controlling the anodization conditions and utilization of nanoimprint technique. By the precise structure design and control, a number of unique nanostructures, including nanopillars, nanotowers, and nanocones, have been successfully fabricated using large-pitch AAMs as nanoengineering templates. This approach can be extended to a variety of other complex structures compatible with diverse materials. Particularly, a-Si nanocones have been fabricated as 3-D nanophotonic structures with characterization of their intriguing optical anti-reflection property. These results directly indicate the potential application of the reported approach for photonics and optoelectronics.

## Competing interests

The authors declare that they have no competing interests.

## Authors’ contributions

QL prepared the AAM templates, carried out the reflectance spectrum of a-Si nanocone arrays by both experiments and simulations, and drafted the manuscript. SFL fabricated a-Si nanocone arrays based on the AAM templates. KHT helped on the fabrication of PC nanostructures based on the AAM templates. BH gave some suggestions on FDTD simulations. ZF provided the idea and completed the manuscript. All authors read and approved the final manuscript.

## Supplementary Material

Additional file 1 **Supporting Information.** The file contains Figure S1 to Figure S5. Click here for file
