# Supplementary material for: Programmable nanoengineering templates for fabrication of three-dimensional nanophotonic structures
Source: Nanoscale Res Lett. 2013 Jun 7;8(1):268. doi: 10.1186/1556-276X-8-268 (PMC3685551; doi:10.1186/1556-276X-8-268)
Supplement: Additional file 1 — Supporting Information. The file contains Figure S1 to Figure S5. [file 1556-276X-8-268-S1.pdf]

Supporting Information

Programmable Nanoengineering Templates for Fabrication of

Three-Dimensional Nanophotonic Structures

Qingfeng Lin, Siu-Fung Leung, Kwong-Hoi Tsui, Bo Hua and Zhiyong Fan*

Department of Electronic and Computer Engineering, Hong Kong University of Science and technology, Clear Water Bay, Kowloon, Hong Kong, China SAR

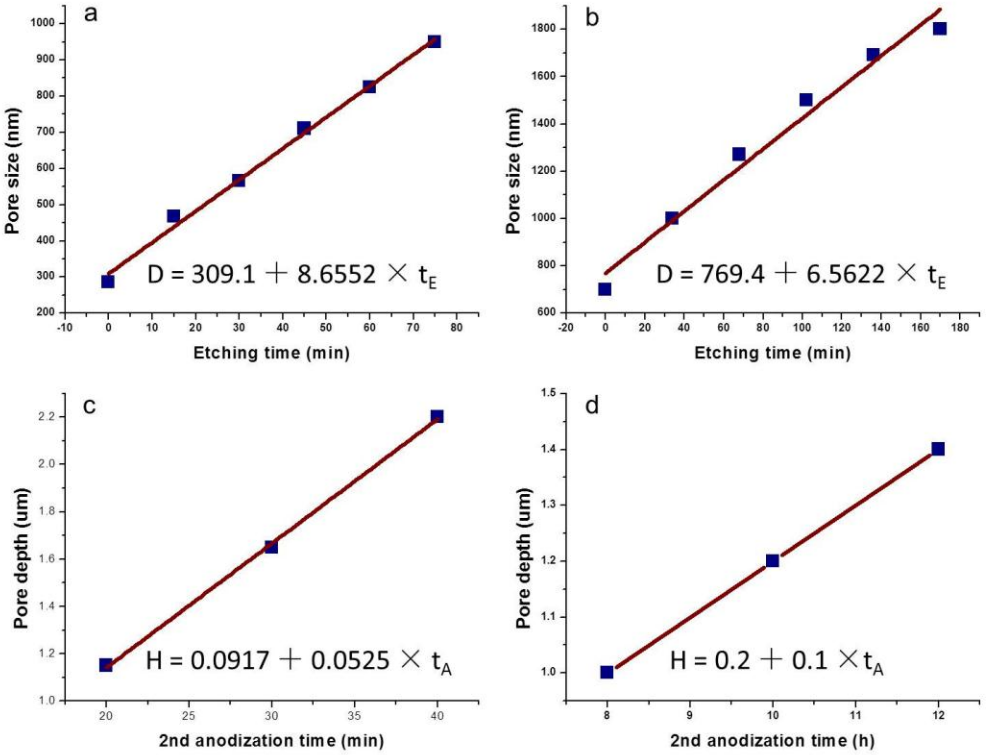

Figure S1. Pore size as a function of wet etching time in 5 wt% H_3PO_4 at 53 ^oC for a) 1 μm pitch and b) 2 μm pitch AAM. Pore depth as a function of 2nd anodization time for c) 1 μm pitch and d) 2 μm pitch AAM.

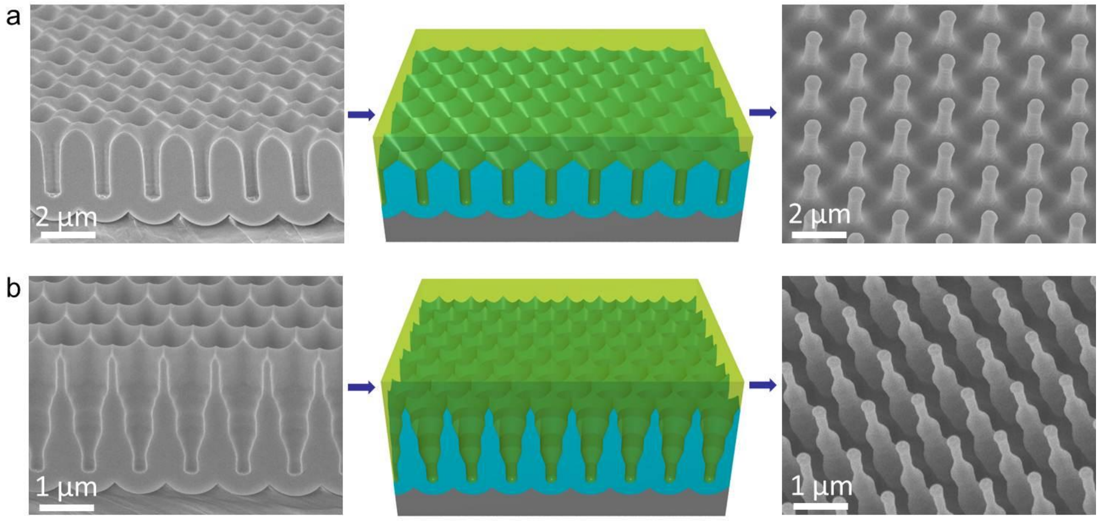

Figure S2. Thermal press process to template a) PC nanopillars and b) PC nanotowers.

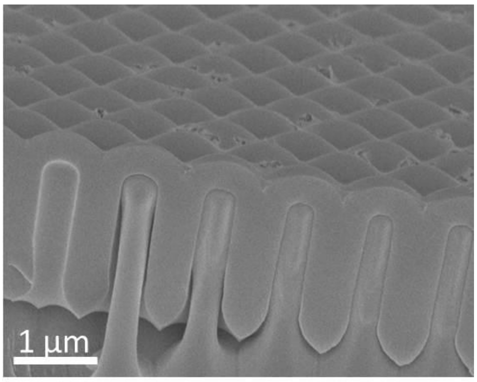

Figure S3. Cross-sectional view SEM image of PI nanopillars, showing that the beads on top of them are formed during peeling process.

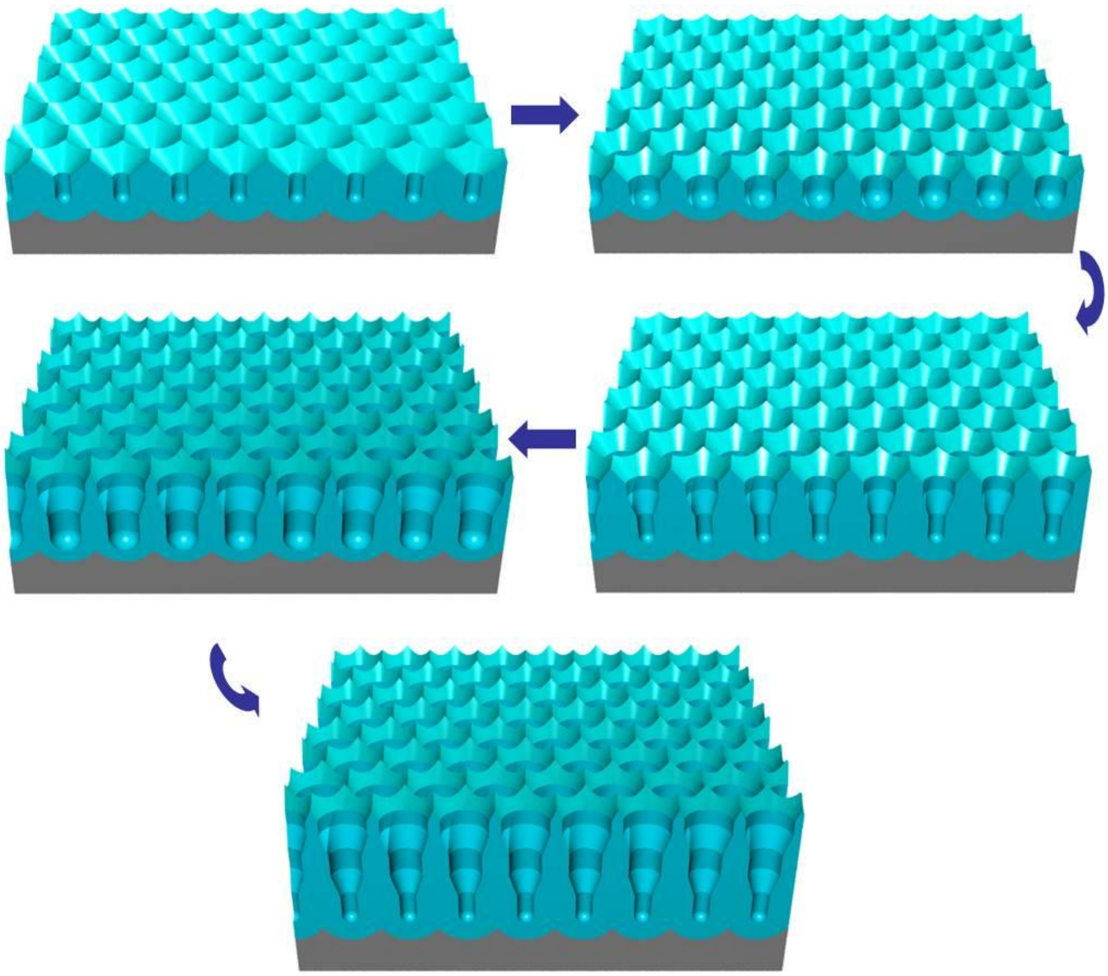

Figure S4. Schematic four-step anodization process for tri-diameter AAM, drawing from 2nd anodization.

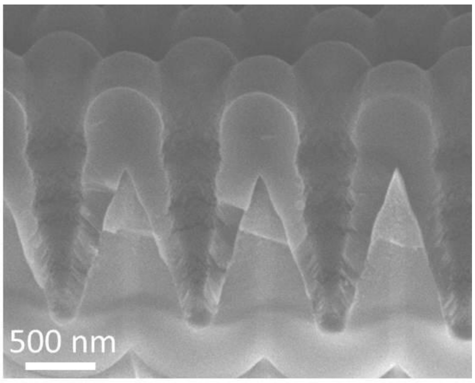

Figure S5. Cross-sectional view SEM image of a-Si nanocones embedded in AAM.
